# The Impact of Stereotype Threat on Technology Usage in Older Adults

**DOI:** 10.1093/geroni/igaf122.3667

**Published:** 2025-12-31

**Authors:** Anna Robison, James Houston

**Affiliations:** University of South Florida, Tennessee, United States; Middle Tennessee State University, Murfreesboro, Tennessee, United States

## Abstract

Negative stereotypes surrounding older adults and technology use, such as incompetency or inability to learn new technologies, continue to permeate American society. These negative stereotypes may serve as a barrier to technology adoption through a concept known as age-based stereotype threat, further contributing to the digital divide and preventing older adults from utilizing technologies that may improve quality of life, independence, and feelings of social isolation. The present study aimed to explore age-based stereotype threat in relation to an objective smartphone use task. The research questions asked if there will be differences between the control and experimental groups on performance on the smartphone task, time to complete the task, and anxiety. We also compared age group differences on the smartphone task. The sample (n = 33) was collected from a community senior center in Tennessee and consisted of older adults aged 55 or older. We divided the sample into those 55-65 (n = 6), 66-75 (n = 13), and 76 + (n = 13) for comparison. We saw no group differences across conditions for the smartphone task, time, or anxiety, but did see a significant difference in the smartphone task by age group, χ² (2) = 11.97, p = .003. Post hoc tests revealed that the 76+ age group scored lower on the smartphone task than other age groups, indicating potential cohort effects. Reasons for no differences may include a lack of pressure on the experimental group and stereotype threat construct validity concerns. Future research should increase difficulty on the smartphone task and explore confounds.

